# Influence of N-acetyltransferase 2 (*NAT2*) genotype/single nucleotide polymorphisms on clearance of isoniazid in tuberculosis patients: a systematic review of population pharmacokinetic models

**DOI:** 10.1007/s00228-022-03362-7

**Published:** 2022-07-19

**Authors:** Levin Thomas, Arun Prasath Raju, Chaithra, Sonal Sekhar M, Muralidhar Varma, Kavitha Saravu, Mithu Banerjee, Chidananda Sanju SV, Surulivelrajan Mallayasamy, Mahadev Rao

**Affiliations:** 1 Department of Pharmacy Practice, Manipal College of Pharmaceutical Sciences, Manipal Academy of Higher Education, Manipal, 576104 Karnataka India; 2 Department of Infectious Diseases, Kasturba Medical College, Manipal Academy of Higher Education, Manipal, 576104 Karnataka India; 3 Department of Biochemistry, All India Institute of Medical Sciences, Jodhpur, 342005 Rajasthan India; 4 District Tuberculosis Control Office, Ajjarakad, 576001 Karnataka India

**Keywords:** Isoniazid, *NAT2*, Population pharmacokinetic, TB

## Abstract

**Purpose:**

Significant pharmacokinetic variabilities have been reported for isoniazid across various populations. We aimed to summarize population pharmacokinetic studies of isoniazid in tuberculosis (TB) patients with a specific focus on the influence of N-acetyltransferase 2 (*NAT2*) genotype/single-nucleotide polymorphism (SNP) on clearance of isoniazid.

**Methods:**

A systematic search was conducted in PubMed and Embase for articles published in the English language from inception till February 2022 to identify population pharmacokinetic (PopPK) studies of isoniazid. Studies were included if patient population had TB and received isoniazid therapy, non-linear mixed effects modelling, and parametric approach was used for building isoniazid PopPK model and *NAT2* genotype/SNP was tested as a covariate for model development.

**Results:**

A total of 12 articles were identified from PubMed, Embase, and hand searching of articles. Isoniazid disposition was described using a two-compartment model with first-order absorption and linear elimination in most of the studies. Significant covariates influencing the pharmacokinetics of isoniazid were *NAT2* genotype, body weight, lean body weight, body mass index, fat-free mass, efavirenz, formulation, CD4 cell count, and gender. Majority of studies conducted in adult TB population have reported a twofold or threefold increase in isoniazid clearance for *NAT2* rapid acetylators compared to slow acetylators.

**Conclusion:**

The variability in disposition of isoniazid can be majorly attributed to *NAT2* genotype. This results in a trimodal clearance pattern with a multi-fold increase in clearance of *NAT2* rapid acetylators compared to slow acetylators. Further studies exploring the generalizability/adaptability of developed PopPK models in different clinical settings are required.

## Introduction

Isoniazid, first synthesized by two Prague chemists, Hans Meyer and Josef Mally, in 1912, was subsequently demonstrated to have antitubercular activity in three different laboratories (Squibb and Hoffmann La Roche in the USA and Bayer in West Germany) in 1951 [1, 2]. Isoniazid has remained the first-line therapy for tuberculosis (TB) even after 70 years of clinical therapy and the centenary of its synthesis [3]. A considerable proportion of TB population has been reported to have significant isoniazid pharmacokinetic variabilities. The pooled proportion of TB patients with low isoniazid concentration after 2 h of intake (*C*_2h_) from 26 studies was reported to be 0.43 (95% CI 0.32–0.55) [4]. Patients with lower isoniazid concentrations were associated with poor treatment outcomes, such as delayed sputum culture conversion [5]. Several covariates such as age, gender, food, drug-drug interactions (DDIs), nutritional status, comorbidities, and N-acetyltransferase 2 (*NAT2*) genotype may account for the pharmacokinetic variability of isoniazid [6].

*NAT2* genotype is one of the most important covariates influencing the plasma concentration of isoniazid [7]. Among the three *NAT2* acetylator phenotypes, rapid acetylators achieve the lowest and slow acetylators achieve the highest plasma concentration of isoniazid [7]. *NAT2* slow acetylator TB patients have been reported to have a comparatively higher early bactericidal activity of isoniazid than rapid acetylators [8]. A metanalysis study of 13 randomized clinical trials reported that *NAT2* rapid acetylators were more likely to have microbiological failure (pooled risk ratio [RR], 2.0; 95% confidence interval [CI], 1.5–2.7), adverse drug reaction (ADR) (RR, 2.0; CI, 1.1–3.4), and relapse (RR, 1.3; CI, 0.9–2.0) than slow acetylators [9]. On the other hand, a pooled analysis of 37 studies reported that slow *NAT2* acetylators had increased the risk for the development of anti-TB drug-induced liver injury (AT-DILI) compared to non-slow *NAT2* acetylators (intermediate and fast *NAT2* acetylators) (overall odds ratio (OR) = 3.15 (95% CI 2.58–3.84, heterogeneity measure (*I*^2^) = 51.3%, *p* = 0.000)) in TB patients [10]. Dose stratification of isoniazid among TB patients based on *NAT2* genotype reduced the incidence of treatment failure among rapid acetylators and DILI among slow acetylators [11]. Hence, it is imperative to design precise dosage regimen for isoniazid to achieve better clinical outcomes and reduce ADRs in TB patients. The population pharmacokinetics (PopPK) approach has emerged as a potential tool for the optimization of antitubercular therapy (ATT) [12].

PopPK estimates drug pharmacokinetic parameters and has emerged as a powerful approach for identifying the sources and correlates of pharmacokinetic variability in a particular patient population [13]. PopPK approach allows several advantages over traditional pharmacokinetics such as permitting sparse sampling approach, cost effectiveness, concentrations without regard to steady-state conditions; allowing irregularly measured concentrations, estimation of variabilities along with identification of its sources; and combining heterogenous types of data from varying sources [14, 15]. PopPK models may undertake a parametric or nonparametric approach [15]. PopPK models using parametric approach have a good explanatory potential and exhibit good flexibility for unusual situations requiring complex models, easier interpretation of covariate effects, and easier for conceptualization and fitting to observations [15, 16].

A systematic review (Vietnamese language) reported ten PopPK studies from inception till July 2017. The selection criteria included articles with non-TB populations, without *NAT2* genotyping, and with all types of modelling strategies [17]. A meta-analysis by Hong et al. evidenced that the dose-normalized summary estimates of isoniazid maximum [peak] plasma drug concentration (*C*_max_) and area under the plasma concentration–time curve (AUC) of the TB population were significantly lower than that of healthy volunteers (*C*_max_ and AUC were 36% and 26% lower in adult TB patients compared to healthy volunteers). This implies that currently recommended isoniazid dosages produced less drug exposure in TB patients compared to healthy volunteers. *NAT2* acetylator status also significantly influenced the isoniazid pharmacokinetic properties [7]. We presume that several isoniazid PopPK studies exploring *NAT2* genotype/single-nucleotide polymorphism (SNP) as a covariate would have been published to address the pharmacokinetic variabilities in isoniazid among different TB populations. Therefore, we performed a systematic review of relevant articles to assess the significance of *NAT2* genotype/SNP on the clearance of isoniazid.

## Methods

### Literature search strategy

The review was conducted according to the Preferred Reporting Items for Systematic Reviews and Meta-Analyses (PRISMA) statement 2020 [18]. A systematic search of PubMed and Embase for articles published in the English language from inception till Feb 3, 2022 was performed to identify PopPK models of isoniazid in TB subjects. Hand searching of articles were also carried out to identify additional relevant articles. Search terms included for the literature search were: “isoniazid” OR “INH” OR “antitubercular” AND “population pharmacokinetic*” OR “PopPK” OR “NONMEM” OR “nonlinear mixed effect* model*” AND “Tuberculosis” OR “TB.”

### Study selection criteria

Two independent reviewers (L.T. and A.P.R.) performed literature search, identification, and selection. The reviewers reviewed the titles, abstracts, and full text of the articles in accordance with the defined inclusion and exclusion criteria. Any disagreements were resolved by a third reviewer (S.M.). Studies were included based on the following criteria: (i) non-linear mixed-effects modelling approach was used for PopPK analysis; (ii) parametric approach was used; (iii) separate model developed for isoniazid in TB patients; and (iv) *NAT2* SNP/genotype was used as a covariate for PopPK model. Conversely, studies that met the following exclusion criteria were removed: (i) model developed for isoniazid other than TB; (ii) study population was non-TB population/latent TB/healthy volunteers, and (iii) isoniazid was given as preventive therapy.

### Data extraction

The full text of selected articles was reviewed, and data were extracted using a standardized extraction form, independently by two authors (L.T. and A.P.R.) and were cross checked. Data discrepancies were resolved by S.M.. For all selected articles, the first author, publication year, total study sample size, gender, samples used for isoniazid modelling, age, body weight, percentage of human immunodeficiency virus (HIV) and diabetes mellitus (DM) population, country of the study population, *NAT2* SNPs investigated, *NAT2* genotype, analytical method, PopPK software, structural model, external validation, PopPK estimates, residual variability, clearance based on *NAT2* SNPs/ genotype, and significant covariates affecting PopPK model were extracted. The extracted data was verified by M.S.R. and M.R. Illustration of PRISMA flow chart was created using Microsoft PowerPoint, and illustrations based on selected studies were performed in RStudio [19] using ggplot2 package [20].

### Data quality assessment

The quality assessment of isoniazid based PopPK studies was carried out using an adopted checklist that was developed from (i) previously published clinical pharmacokinetics [21], (ii) population pharmacokinetic-pharmacodynamic guidelines [22], and (iii) two studies that developed a combination of the (i) and (ii) checklist [23, 24]. The combined modified checklist consisted of 46 criteria categorized into five domains: the title, abstract, background/introduction, methods/results, and discussion/conclusion, as shown in Table 1. A score of 1 was given for each criterion, if the relevant information was identified from the study, else zero point was given. All the 12 isoniazid PopPK studies were assessed based on these criteria. The compliance rate of each study was calculated using the following equation and reported in percentage.

**Table 1:** Checklist for assessing the quality of isoniazid PopPK studies

Quality criteria (46)	Soedarsono et al. 2022 [25]	Gao et al. 2021 [26]	Cho et al. 2021 [27]	Jing et al. 2020 [28]	Sundell et al. 2020 [29]		Huerta-García et al. 2020 [30]	Sekaggya-Wiltshire et al. 2019 [31]	Naidoo et al. 2019 [32]	Denti et al. 2015 [33]	Panjasawatwong et al. 2020 [34]	Horita et al. 2018 [35]	Abdelwahab et al. 2020 [36]	Total compliance rate of each criterion (%)
**Title**														
The title identifies the drug(s) and patient population(s) studied	✔	×	✔	✔	✔		✔	✔	✔	✔	✔	✔	✔	91.6
**Abstract**														
Name of the drug(s) studied	✔	✔	✔	✔	✔		✔	✔	✔	✔	✔	✔	✔	100
Patient population studied	✔	✔	✔	✔	✔		✔	✔	×	✔	✔	✔	✔	91.6
Primary objective(s)	✔	✔	✔	✔	✔		✔	✔	✔	✔	✔	✔	✔	100
Major findings	✔	✔	✔	✔	✔		✔	✔	✔	✔	✔	✔	✔	100
**Background/introduction**														
Study rationale	✔	✔	✔	✔	✔		✔	✔	✔	✔	✔	✔	✔	100
Specific objectives/hypothesis	✔	✔	✔	✔	✔		✔	✔	✔	✔	✔	✔	✔	100
**Methods/results**														
Ethics approval	✔	✔	✔	✔	✔		✔	✔	✔	✔	✔	✔	✔	100
Eligibility criteria of study participants	✔	✔	✔	✔	✔		✔	✔	✔	✔	✔	✔	✔	100
Co-administration of food	NA	NA	✔	✔	NA		NA	NA	NA	✔	NA	NA	NA	100
Co-administration of drug	×	NA	×	NA	✔		×	NA	✔	×	NA	NA	✔	42.8
Dosing	✔	✔	✔	✔	✔		✔	✔	✔	✔	✔	✔	✔	100
Frequency	✔	✔	✔	✔	✔		✔	✔	✔	✔	✔	✔	✔	100
Route of administration/formulation	✔	✔	✔	✔	✔		✔	✔	✔	✔	✔	✔	✔	100
Sampling time and frequency	✔	✔	✔	✔	✔		✔	✔	✔	✔	✔	✔	✔	100
Type of sample for quantitative drug measurement mentioned (whole blood/plasma/CSF/other)	✔	✔	✔	✔	✔		✔	✔	✔	✔	✔	✔	✔	100
Quantitative bioanalytical methods and validation used in the study are referenced or described	✔	✔	✔	✔	✔		✔	✔	✔	✔	✔	✔	✔	100
Statistical method and software used (if applicable)	NA	NA	NA	NA	NA		NA	NA	NA	NA	NA	✔	NA	100
Modelling software and version used	✔	✔	✔	✔	✔		✔	✔	✔	✔	✔	✔	✔	100
Modelling assumptions made	✔	✔	✔	✔	✔		✔	✔	✔	✔	×	×	✔	83.3
Estimation method(s) used	✔	✔	✔	✔	✔		✔	✔	✔	✔	✔	✔	✔	100
Structural model	✔	✔	✔	✔	✔		✔	✔	✔	✔	✔	✔	✔	100
Covariates tested	✔	✔	✔	✔	✔		✔	✔	✔	✔	✔	✔	✔	100
Covariate analysis strategy	✔	✔	✔	✔	✔		✔	✔	✔	✔	✔	✔	✔	100
Residual error model	✔	✔	✔	✔	✔		✔	✔	✔	✔	✔	✔	✔	100
The specific body weight used in drug dosing and pharmacokinetic calculations are reported (i.e., ideal body weight/actual body weight/adjusted body weight)	** × **	×	✔	×	×		×	✔	✔	✔	×	×	✔	41.6
Methods for final model evaluation	✔	✔	✔	✔	✔		✔	✔	✔	✔	✔	×	✔	91.6
External model validation	×	✔	✔	×	×		✔	×	×	×	×	×	×	25
Model selection criteria (OFV/AIC etc.)	✔	✔	✔	✔	✔		✔	✔	✔	×	✔	✔	✔	91.6
Number of study subjects	✔	✔	✔	✔	✔		✔	✔	✔	✔	✔	✔	✔	100
Number of samples used for analyses	✔	✔	✔	✔	✔		✔	✔	✔	✔	✔	✔	✔	100
Number of BLOQ samples (if applicable)	×	✔	NA	✔	✔		NA	✔	×	✔	✔	✔	✔	80
Details of missing data mentioned (if applicable)	✔	NA	✔	NA	✔		NA	✔	✔	✔	✔	✔	✔	100
Handling of missing data (if applicable)	✔	NA	×	NA	✔		NA	×	×	✔	✔	×	✔	55.5
Handling of BLOQ/outliers (if applicable)	✔	✔	NA	✔	✔		NA	✔	✔	×	✔	✔	✔	90
Equations for all model structures and covariate relationships	×	×	✔	×	×		×	×	✔	×	×	×	×	20
Detailed descriptions of planned simulations (if applicable)	NA	✔	✔	✔	✔		✔	✔	NA	NA	✔	✔	NA	100
Demographics details and clinical variables	✔	✔	✔	✔	✔		✔	✔	✔	✔	✔	✔	✔	100
Plot of concentration vs time/effects	×	×	×	✔	×		×	×	×	×	×	×	×	8.3
Schematic of the final model	×	×	×	×	✔		×	×	×	×	×	×	✔	16.6
Table of the final model parameters	✔	✔	✔	✔	✔		✔	✔	✔	✔	✔	✔	✔	100
Summary of the model building process and the derived final model	✔	✔	✔	✔	✔		✔	✔	✔	✔	✔	✔	✔	100
Final model evaluation plots	✔	✔	✔	✔	✔		✔	✔	✔	✔	✔	✔	✔	100
A description of simulation results or scenarios (if applicable)	NA	✔	✔	✔	✔		✔	✔	NA	NA	✔	✔	NA	100
**Discussion/conclusion**														
Study limitations	✔	✔	✔	✔	✔		✔	✔	✔	✔	✔	✔	✔	100
Study findings	✔	✔	✔	✔	✔		✔	✔	✔	✔	✔	✔	✔	100
**Total compliance rate of each study (%)**	83.3	87.8	90.6	90.4	90.9		87.5	88.3	85.7	83.7	86.0	81.8	92.8	-

✔ Denotes study reported the quality criteria; × denotes study did not report the quality criteria; *AIC*, Akaike’s information criterion; *BLOQ*, below limit of quantification; *CS*, cerebrospinal fluid; *NA*, not applicable; *NAT2*, N-acetyltransferase 2; *OFV*, objective function value

$$\mathrm{Compliance\;rate}\left(\mathrm{\%}\right)=\frac{\mathrm{Total\;number\;of\;criteria\;met}}{\mathrm{Total\;number\;of criteria\;that\;are\;applicable\;for\;the\;study}}\times 100$$

## Results

### Literature search

**Fig. 1:**
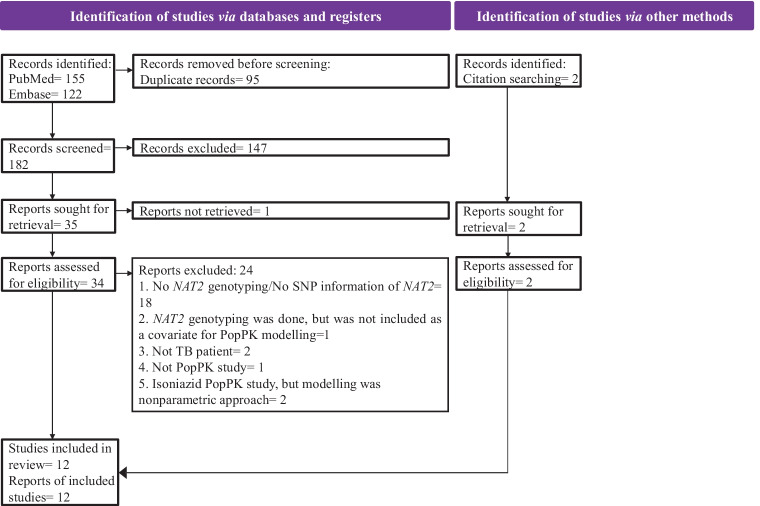
PRISMA flow diagram showing the literature search and selection of isoniazid PopPK studies in TB patients. *NAT2* N-acetyltransferase 2, *PopPK* population pharmacokinetics, *SNP* singlenucleotide polymorphism, *TB* tuberculosis

A total of 155, 122, and 2 articles were identified from PubMed, Embase, and hand searching of articles, respectively. A total of 95 duplicate articles were removed. After title and abstract screening, 37 articles were available for full texts. Among these 37 articles, 25 articles were excluded for the following reasons, including (i) articles did not have *NAT2* genotype or no SNP information of *NAT2* genotype was available (*n* = 18), (ii) *NAT2* genotype was carried out; however, it was not included as a covariate for isoniazid PopPK modelling building (*n* = 1), (iii) the patient population of the study was not having TB (*n* = 2), (iv) full text of the articles was not available (*n* = 1), (v) was not a PopPK study (*n* = 1), and (vi) nonparametric approach was used for isoniazid PopPK modelling (*n* = 2). A total of 12 articles remained for the systematic review. The PRISMA flow diagram detailing the selection of PopPK studies of isoniazid for the systematic review is shown in Fig. 1.

### Quality assessment of selected literatures

All the studies had a compliance rate of above 80% for the quality of the PopPK study (range: 81.8–92.8%). Criterions with minimum compliance were a plot of concentration versus time/effects (8.3%), schematic of the final model (16.6%), equations for all model structures and covariate relationships (20%), external validation (25%), information regarding the specific body weight used in drug dosing and pharmacokinetic calculations (*n* = 41.6%), and co-administration of drugs (42.8%) as shown in Table 1.

### Study population and sample size

Of the identified 12 studies, nine were carried out in adult TB population [25–33], two in pediatric TB [34, 35] and one in pregnant TB population [36]. Five studies were conducted in Asian countries, of which two were from China [26, 28], one each from Indonesia [25], Korea [27], and Vietnam [34]. Six studies were from African continent, with two studies from South Africa [32, 36] and one study each from Rwanda [29], Uganda [31], Ghana [35], and Tanzania [33]. One study was conducted in the Mexican population [30]. The number of TB patients and total number of samples with isoniazid ranged from 29 to 454 and 141 to 1814 respectively as shown in Table 2. Fifty percent of studies had less than or equal to hundred TB patients.

**Table 2:** Baseline demographic and genotype data of all the isoniazid PopPK studies in TB patients

Study, year published [reference]	TB population	Study sample (gender: males/females)	Total samples for isoniazid	Age	Body weight	HIV-Yes (%)	DM-Yes (%)	*NAT2* SNPs investigated	*NAT2* genotype % of RA/IA/SA
**Adult TB **									
Soedarsono et al. 2022 [25]	Indonesian	107 (63/44)	153	Median (range) = 43 (18–77)	Median (range) = 50 (32–82)	7.4	21.5	c.191G > A c.282C > T c.341 T > C c.590G > A c.803A > G c.857G > A	RA = 14.9 IA = 45.8 SA = 39.2
Gao et al. 2021 [26]	Chinese	Model building group = 217 (147/70) Validation group = 61 (41/20)	Model building group = 1230 Validation group = 305	Mean (± SD) = Model building group-41(± 10.6) (Jiangsu = 40.1 (± 11.1) Sichuan = 40.2 (± 10.8) Fujian = 40.4 (± 11.2) Shandong = 41.9 (± 9.8) Validation group = 40.8 (± 11.1)	Mean (± SD) = Model building group-Jiangsu = 51.4 (± 10.1) Sichuan = 51.9 (± 9.4) Fujian = 51.8 (± 9.2) Shandong = 53.4 (± 10.1) Validation group = 51.6 (± 9.0)	- (Exclusion)	Model building group = 15.2 Validation group = 11.5	c.282C > T c.341 T > C c.481C > T c.590G > A c.803A > G c.857G > A	Model building group- (*n* = 217): RA = 46.1 IA = 32.2 SA = 21.7 Validation group (*n* = 61): RA = 45.9 IA = 39.3 SA = 14.8
Cho et al. 2021 [27]	Korean	Model building group = 363 (255/106)* Validation group = 91 (48/43)	Total 477 (Model building group: Validation group = 4:1)	Mean (± SD): Model building group = 55.7 (17.2) Validation group = 54.4 (18.0)	Mean (± SD): Model building group = 60.9 (11.7) Validation group = 56.5 (10.9)	-	Model building group = 83.7 Validation group = 86.8	c.191G > A c.282C > T c.341 T > C c.590G > A c.803A > G c.857G > A	Model building group (*n* = 363) RA = 39.4 IA = 48.8 SA = 10.7 UN = 1.1 Validation group (*n* = 91) RA = 45.1 IA = 38.1 SA = 15.4 UN = 1.0
Jing et al. 2020 [28]	Chinese	89 (59/30)	195	Mean (± SD) = 42.9 (15.6)	Mean (± SD) = 60.0 (12.3)	-	-	c.341 T > C c.481C > T c.590G > A c.857G > A	RA = 36.0 IA = 42.7 SA = 21.3
Sundell et al. 2020 [29]	Rwandan	63 (37/26)	432	Median (range): Concurrent HIV treatment = 40 (26–57) HIV treatment naïve = 38 (21–52)	Median (range): Concurrent HIV treatment = 48 (35–65) HIV treatment naïve = 50 (30–68)	100	-	c.282C > T c.341 T > C c.481C > T c.590G > A c.803A > G c.857G > A	RA = 8 IA = 48 SA = 44
Huerta-García et al. 2020 [30]	Mexican	Model building group = 55 (31/24) Validation group = 14 (5/9)	Model building group = 294 Validation group = 91	Mean (± SD): Model building group = 44.7 (16.9) Validation group = 48.5 (14.7)	Mean (± SD): Model building group = 56.6 (14.5) Validation group = 58.9 (12.4)	- (Exclusion)	Model building group = 27.3 Validation group = 28.6	c.282C > T c.341 T > C c.481C > T c.590G > A c.803A > G c.857G > A	Model building group (*N* = 55): RA = 18.2 IA = 47.3 SA = 34.5 Validation group (*N* = 14): RA = 21.4 IA = 42.9 SA = 35.7
Sekaggya-Wiltshire et al. 2019 [31]	Ugandan	254 (148/106)	1814 (251)	Median of 254 patients (IQR) = 35 (29, 40)	Median of 254 patients (IQR) = 52 (47.5, 59)	100	-	c.590G > A (rs1799930)	*NAT2* rs1799930 GG = 41.2 *NAT2* rs1799930 GA = 37 *NAT2* rs1799930 AA = 3.5 UN = 17.7
Naidoo et al. 2019 [32]	South African	172 (119/53)	573	Median (range) = 35 (30–41)	Median (range) = 55.7 (50.3–62.1)	73.8	-	c.191G > A c.341 T > C c.590G > A c.857G > A	RA = 18 IA = 43 SA = 34
Denti et al. 2015 [33]	Tanzanian	100 (58/42)	574	Median (IQR) = 35 (29; 40)	Median (IQR) = 51.9 (48.3; 57.3)	50	-	c.282C > T c.341 T > C c.481C > T c.590G > A c.803A > G c.857G > A	RA = 2 IA = 48 SA = 48 UN = 2
**Pediatric TB**									
Panjasawatwong et al. 2020 [34]	Vietnamese	100 (56/44)	523 plasma and 140 CSF samples	Median (minimum–maximum) = 3.0 (0.167 to 15.0)	Median (minimum–maximum) = 10.9 (4.0 to 43)	4	-	c.191G > A c.282C > T c.341 T > C c.481C > T c.590G > A c.803A > G c.857G > A	RA = 17 IA = 47 SA = 28 UN = 8
Horita et al. 2018 [35]	Ghanaian	113 (63/50)	561	Median (IQR) = 5.00 (2.17–8.25)	Median (IQR) = 14.3 (9.70–20.1)	52.2	-	c.191G > A c.341 T > C c.590G > A c.857G > A	RA = 10.6 IA = 44.2 SA = 45.1
**Pregnant TB**									
Abdelwahab MT et al. 2020 [36]	South African	29	141 (77 during pregnancy and 64 postpartum)	Median (IQR) = 28.1 (25.2–29.9)	Median (IQR): Prepartum = 66.0 (60.0–80.0) Postpartum = 63.5 (57.3–72.8)	100	-	c.191G > A c.341 T > C c.590G > A c.857G > A	RA = 10 IA = 34 SA = 38 UN = 17

*CSF* Cerebrospinal fluid, *DM* Diabetes mellitus, *HIV* Human immunodeficiency virus, *IA* Intermediate acetylators, *IQR* Interquartile range, *NAT2* N-acetyltransferase 2, *RA* Rapid acetylators, *SA* Slow acetylators, *SD* Standard deviation, *SNP* Single nucleotide polymorphisms, *TB* Tuberculosis, *UN* Unknown

^*^2 was unknown gender

### Sampling procedure

Some of the studies have taken minimal number of samples from TB patients, whereas few others have taken five or more samples from a patient on a single day and/or different days. The sampling involved blood withdrawal at random time intervals and/or at fixed time intervals. PopPK study by Soedarsono et al. involved one-time blood collection from outpatients and up to two times from inpatients. Jing et al. developed a PopPK model using 195 samples from 89 patients, which involved one-time, two-time, three-time, and four-time blood sample collections from 1, 71, 16, and 1 TB patients respectively. PopPK model of Sekaggya-Wiltshire et al. involved collection of blood samples at 1, 2, and 4 h after ATT intake, at second, eighth, twelfth, and twenty-fourth weeks after ATT initiation accounting to 1814 samples from 251 patients. A study by Gao et al. involved blood collection at pre-dose and at first, second, fourth, sixth, and eighth hour after ATT administration.

### Comorbidities

Eight out of the 12 studies had TB patients coinfected with HIV infection (range: 4–100%). PopPK models by three studies had all the TB patients with HIV infection [29, 31, 36]. However, only four out of the 12 studies have reported the status of DM in TB patients [25–27, 30].

### *NAT2* SNP(s) and genotype

**Fig. 2:**
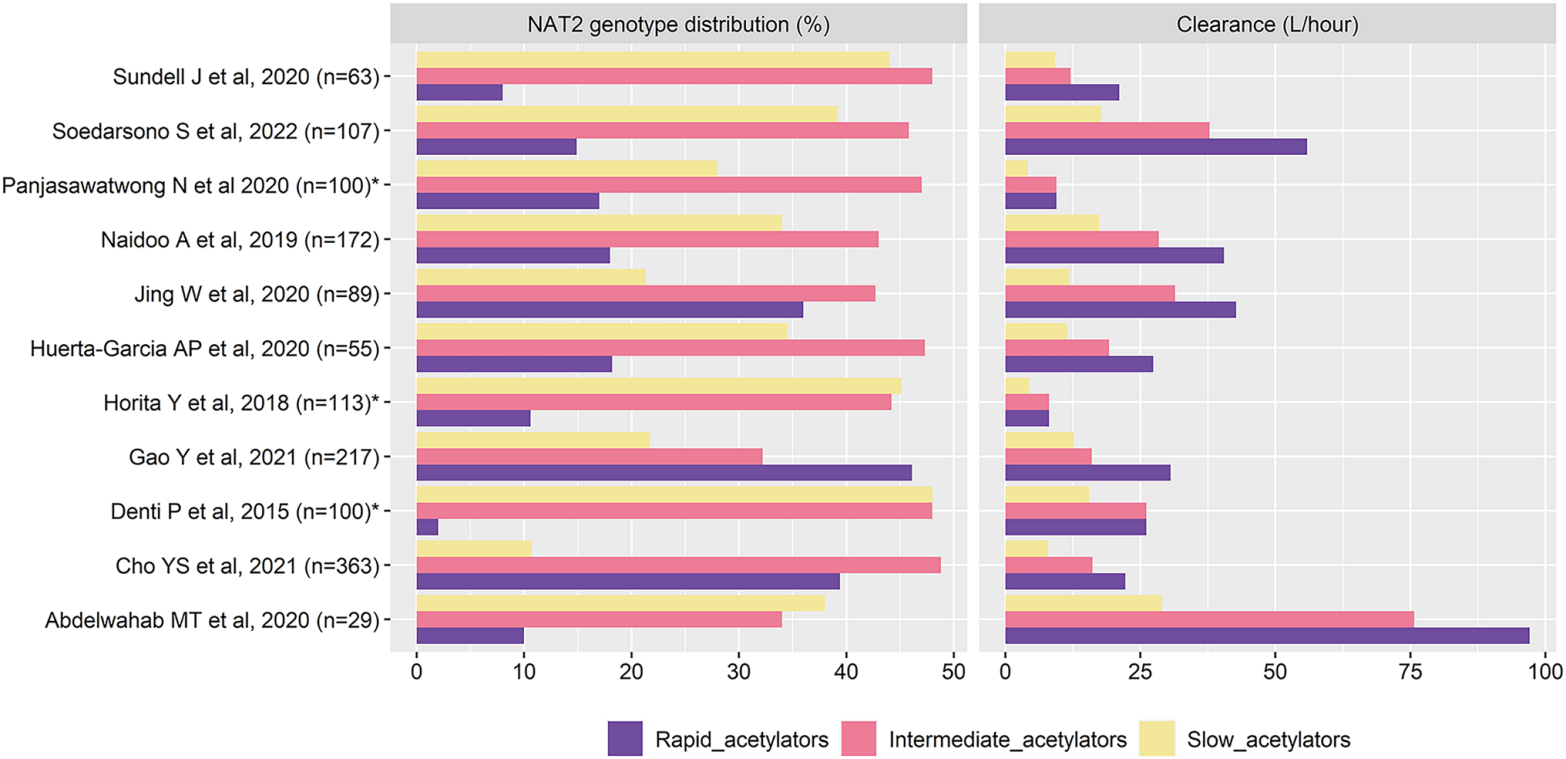
Bar chart integrating *NAT2* genotype distribution and corresponding clearance across isoniazid PopPK studies. *NAT2* N-acetyltransferase 2. *The clearance of isoniazid for *NAT2* rapid and intermediate acetylators were clubbed as one category. The proportion of TB patients with unknown genotype in any study is not represented in the bar chart

A study by Sekaggya-Wiltshire et al. who assessed only single SNP (c.590G > A), was excluded from comparison with other studies based on genotype; all other studies had evaluated the *NAT2* genotype using different combinations of SNP panels. A study by Panjasawatwong et al. derived the *NAT2* genotype for Vietnamese TB children using the seven SNP panels of c.191G > A, c.282C > T, c.341 T > C, c.481C > T, c.590G > A, c.803A > G, and c.857G > A. Four studies (Gao et al. Sundell et al. Huerta-García et al. and Denti et al.) derived *NAT2* genotype using the six SNP panels of c.282C > T, c.341 T > C, c.481C > T, c.590G > A, c.803A > G, and c.857G > A, whereas two studies (Soedarsono et al. and Cho et al.) derived *NAT2* genotype using the six SNP panels of c.191G > A, c.282C > T, c.341 T > C, c.590G > A, c.803A > G, and c.857G > A. Three studies (Naidoo et al. Abdelwahab et al. and Horita et al.) used the four SNP panels of c.191G > A, c.341 T > C, c.590G > A, and c.857G > A, and one study (Jing et al.) employed the four SNP panels of c.341 T > C, c.481C > T, c.590G > A, and c.857G > A to derive the *NAT2* genotype. The percentage of *NAT2* rapid acetylators ranged from 2 to 46.1%, whereas of *NAT2* intermediate and slow acetylators from 32.2 to 48.8% and 10.7 to 48% respectively, as shown in the Fig. 2.

### Bioanalytical methods

Liquid chromatography with tandem mass spectrometry (LC–MS/MS) was the most preferred analytical method to determine the isoniazid concentration in the samples (*n* = 10). On the other hand, Huerta-García et al. and Sekaggya-Wiltshire et al. used high-performance liquid chromatography (HPLC) and high-performance liquid chromatography-ultraviolet (HPLC–UV), respectively, as shown in Table 3.

**Table 3:** PopPK modelling details of all the isoniazid PopPK studies in TB patients

Study, year published [reference]	Analytical method	Modelling software	Structural model	External validation	PopPK estimates	Residual variability	CL/F values based on *NAT2* (L/hour)	Significant covariates affecting model	Clearance equation
**Adult TB **									
Soedarsono et al. 2022 [25]	LC–MS/MS	NONMEM	One- compartment model with first-order absorption and elimination	No	*V*_*d*_ = 40.5 L *K*_*a*_ = 0.25 h^−1^ BSV of CL/F (%) = 68 (fixed for other parameters but not mentioned)	Additive error mcg/ml = 0.174	RA = 55.9 IA = 37.8 SA = 17.7	BW, *NAT2* genotype	Cl/F_SA_ = 17.7*(BW/50)^0.75^ Cl/F_IA_ = 17.7*(1 + 1.14)*(BW/50)^0.75^ Cl/F_RA_ = 17.7*(1 + 2.16)*(BW/50)^0.75^
Gao et al. 2021 [26]	LC–MS/MS	Phoenix NLME	Two-compartment model with first-order absorption and elimination	Yes	*V*_1_ = 21.2 L *V*_2_ = 125.8 L *K*_*a*_ = 0.68 h^−1^ *Q* = 8.7 L/hour BSV of CL/F (%CV) = 60.9 BSV of *V*_1_ (%CV) = 21.7 BSV of *K*_*a*_ (%CV) = 23.6	Additive error mg/L = 0.178	RA = 30.6 IA = 16.0 SA = 12.6	BW, *NAT2* genotype	Cl/F_SA_ = 12.6*(BW/50)^0.55^ Cl/F_IA_ = 16.0*(BW/50)^0.55^ Cl/F_RA_ = 30.6*(BW/50)^0.55^
Cho et al. 2021 [27]	LC–MS/MS	NONMEM	Two-compartment model with absorption lag time and sequential zero-order and first-order absorption with first-order elimination	Yes	*V*_1_ = 16.5 L *V*_2_ = 36.8 L *K*_*a*_ = 1.21 h^−1^ *Q* = 18.4 L/hour D0 = 0.47 h *t*_lag_ = 0.02 (FIX) hour BSV of CL/F = 0.14 BSV of *V*_1_ = 0.03 (FIX) BSV of *V*_2_ = 0.15(FIX) BSV of *K*_*a*_ = 0.60 (FIX) BSV of D0 = 0.2 (FIX) BSV of *t*_lag_ = 0.22 (FIX)	Proportional error = 0.292 Additive error mcg/ml = 0.134	RA = 22.2 IA = 16.1 SA = 7.9	LBW, *NAT2* genotype	Cl/F_RA_ = 22.2*(LBW/50)^0.75^ Cl/F_IA_ = 22.2*(1 + (-0.274))*(LBW/50)^0.75^ Cl/F_SA_ = 22.2*(1 + (-0.646))*(LBW/50)^0.75^
Jing et al. 2020 [28]	LC–MS/MS	NONMEM	Two-compartment model with first-order absorption and elimination	No	*V*_1_ = 21.1 L *V*_2_ = 27.7 L *K*_*a*_ = 1.70 h^−1^ *Q* = 43.7 L/hour BSV of CL/F (%) = 25.6 BSV of *K*_*a*_ (%) = 75.8 BSV of *Q* (%) = 63.8	Exponential error % = 25.1	RA = 42.7 IA = 31.4 SA = 11.9	CW, *NAT2* genotype	Cl/F_IA_ = 31.4*(CW/58)^0.930^ Cl/F_SA_ = 31.4*(CW/58)^0.930^*0.378 Cl/F_RA_ = 31.4*(CW/58)^0.930^*1.36
Sundell et al. 2020 [29]	LC–MS/MS	NONMEM	Two-compartment model including first-order absorption with one transit compartment and first order elimination	No	*V*_1_ = 41.3 L *V*_2_ = 42.8 L *Q* = = 10.8 L/hour *F* = 1 FIXED MTT = 0.58 h BSV of CL/F (%) = 82.7 BSV of *Q* (%) = 120.6 BSV of F (%) = 27.2 BSV of MTT (%) = 180.6	Proportional error = 0.34	RA = 21.1 IA = 12.1 SA = 9.2	CD4 cell count, gender, *NAT2* genotype	Cl/F_SA_ = 9.2*(BW/50)^0.75^ Cl/F_IA_ = 9.2*(1 + 0.32)*(BW/50)^0.75^ Cl/F_RA_ = 9.2*(1 + 1.29)*(BW/50)^0.75^
Huerta-García et al. 2020 [30]	HPLC	NONMEM	Two compartment open model with first-order rate constant of absorption and elimination	Yes	*V*_1_ = 1.5 L *V*_2_ = 3.8 L *K*_*a*_ = 2.0 h^−1^ *Q* = 9.9 L/hour BSV of CL/F (%) = 47.0 BSV of *V*_1_ (%) = 59.4 BSV of *V*_2_ (%) = 114.0 BSV of *K*_*a*_ (%) = 113.6	Proportional error % = 42.9	RA = 27.4 IA = 19.2 SA = 11.4	BMI, *NAT2* genotype	Cl/F_SA_ = 11.4 Cl/F_IA_ = 19.2 Cl/F_RA_ = 27.4
Sekaggya-Wiltshire et al. 2019 [31]	HPLC–UV	Monolix	Two-compartment disposition with first-order elimination and first-order absorption with a lag time	No	*V*_1_ = 64.1 L *V*_2_ = 46.3 L *K*_*a*_ of RHZE, HR1, HR2 = 1.73 h^−1^ K_a_ of HR3 = 1.64 h^−1^ *Q* = 27.6 L/hour *t*_lag_ = 0.25 (FIX) hour *F* = Reference formulation, RHZE = 1 FIXED HR1% = 1.269 HR2% = 0.848 HR3% = 1.193 BSV of CL/F (%CV) = 53.7 BSV of *V*_1_ (%CV) = 33.8 BSV of *K*_*a*_ (%CV) = 73.1 BSV of *t*_lag_ (%CV) = 9.5 BSV of F (%CV) = 31.8	Proportional error % = 30.6 Additive error mg/l = 0.172	*NAT2* rs1799930 GG carrier (mean) = 22.8 Effect of *NAT2* rs1799930 GA on CL/F (%) = − 26.3 (36), 16.8 mean Effect of *NAT2* rs1799930 AA on CL/F (%) = − 74.6 (28), 5.79 mean Effect of Efavirenz on CL/F(%) = + 24.1(27), 28.29 mean	BW, *NAT2* SNP, efavirenz, formulation	Cl/F_rs1799930 GG_ = 22.8*(BW/52)^0.75^ Cl/F _rs1799930 GA_ = 22.8*(BW/52)^0.75^*(− 26.3%) Cl/F_rs1799930 AA_ = 22.8*(BW/52)^0.75^*(− 74.6%) Effect of efavirenz on isoniazid Cl/F = 22.8*(BW/52)0.75*(+ 24.1%)
Naidoo et al. 2019 [32]	LC–MS/MS	NONMEM	Two-compartment disposition with first-order elimination and first-order absorption with a lag time	No	*V*_1_ = 73.4 L *V*_2_ = 19.8 L *K*_*a*_ = 3.9 h^−1^ *Q* = 1.1 L/hour *F* = 1 FIXED *t*_lag_ = 0.13 BSV of CL/F (%) = 26.3	Proportional error % = 27.8 Additive error mg/l = 0.004 FIXED	RA = 40.5 IA = 28.4 SA = 17.4	*NAT2* genotype, FFM	Cl/F_RA_ = 40.5*(FFM/47)^0.75^ Cl/F_IA_ = 28.4*(FFM/47)^0.75^ Cl/F_SA_ = 17.4*(FFM/47)^0.75^
Denti et al. 2015 [33]	LC–MS/MS	NONMEM	Two-compartment model with transit compartment absorption and first-order elimination	No	*V*_1_ = 48.2 L *V*_2_ = 16.5 L *Q* = 16.1 L/hour *F* = 1 FIXED MTT = 0.924 h Number of transit compartment– “NN” = 2.73 BSV of CL/F (%CV) = 30.7 BSV of F (%CV) = 12.8 BSV of MTT (%CV) = 37.4	Proportional error % = 13.3 Additive error mg/L = 0.0224 FIXED	RA/IA = 26.1 SA = 15.5	FFM, *NAT2* genotype	Cl/F_RA/IA_ = 26.1*(FFM/43)^0.75^ Cl/F_SA_ = 15.5*(FFM/43)^0.75^
**Pediatric TB**									
Panjasawatwong et al. 2020 [34]	LC–MS/MS	NONMEM	Two-compartment model with two fixed-transit absorption compartments	No	*V*_1_ = 3.78 L *V*_2_ = 15.3 L *Q* = 28.0 L/hour MTT = 0.878 h MAT50 = 12.7 months Hill coefficient = 4.7 BSV of CL/F (%CV) = 36.8 BSV of *Q* (%CV) = 101	Additive error mcg/L = 0.474	RA/IA = 9.4 SA = 4.1	BW, post menstrual age, *NAT2* genotype	NA
Horita et al. 2018 [35]	LC–MS/MS	Monolix	Two-compartment model with first-order absorption and linear elimination	No	*V*_1_ = 16.6 L *V*_2=_ 1.07 L *K*_*a*_ = 4.23 h^−1^ *Q* = 8.46 L/hour BSV of CL/F_slow_ (estimate (%CV) = 0.324 (33.3) BSV of CL/F_nonslow_ = 0.48 (50.9) BSV of *V*_1_ (estimate (%CV) = 0.241 (24.5) BSV of *V*_2_ (estimate (%CV) = 1.9 (599.7) BSV of *K*_*a*_ (estimate (%CV) expand = 0.567 (61.6) BSV of *Q* (estimate (%CV) = 0.637 (70.7)	Proportional error = 0.193 Additive error = 0.0393	RA & IA = 8.0 SA = 4.4	BW, *NAT2* genotype	Cl/F_SA_ = 4.44*(Allometric scaling to BW)^0.75^ Cl/F_Nonslow_ = 8.08*(Allometric scaling to BW)^0.75^
Abdelwahab et al. 2020 [36]	LC–MS/MS	NONMEM	Two-compartment disposition model with first-order elimination and transit compartments absorption	No	*V*_1_ = 130 L *V*_2_ = 28.5 L *Q* = 12.4 L/hour MTT = 1.21 h Number of transit compartment– “NN” = 8.01 BSV of CL/F (%CV) = 12.7	Proportional error (%) = 22.2 Additive error mg/l = 0.045	RA = 97.1 IA = 75.7 SA = 29.0	FFM, BW, *NAT2* genotype	Cl/F_RA_ = 97.1*(FFM/FFM_Med_)^0.75^ Cl/F_IA_ = 75.7*( FFM/FFM_Med_)^0.75^ Cl/F_SA_ = 29*( FFM/FFM_Med_)^0.75^ Where FFM_Med_ = 40.0 for post-partum and 41.4 for prepartum

*BMI* Body mass index, *BSV* Between subject variability, *CI* Confidence interval, *CL/F* Apparent total body clearance of drug from plasma after oral administration, *CL/F*_*IA*_ Apparent total body clearance of drug from plasma after oral administration of intermediate acetylators, *CL/F*_*nonslow*_ Apparent total body clearance of drug from plasma after oral administration of non-slow acetylators, *CL/F*_*RA*_ apparent total body clearance of drug from plasma after oral administration of rapid acetylators, *CL/F*_*SA*_ apparent total body clearance of drug from plasma after oral administration of slow acetylators, *CV* coefficient of variation, *CW* current weight, *D0* zero-order absorption rate, *E* ethambutol, *F* bioavailability, *FFM* fat-free mass, *FFM*_*med*_ median fat-free mass, *H *isoniazid, *HPLC* high-performance liquid chromatography, *HPLC–UV*, high-performance liquid chromatography-ultraviolet, *HR1* manufactured by Cosmos Pharmaceutical Limited, *HR2* manufactured by Strides Arco Labs, *HR3* manufactured by Svizera Labs, *IA* intermediate acetylators, *K*_*a*_ absorption rate constant (first-order), *L* liter, *LBW* lean body weight, *LC–MS/MS* liquid chromatography with tandem mass spectrometry, *MAT50* the postmenstrual age at which 50% maturation of clearance occurred, *mg* milligram, *mcg* microgram, *MTT* mean transit time, *NA* not available, *NAT2* N-acetyltransferase 2, *PopPK* population pharmacokinetic, *Q* apparent intercompartmental clearance, *RA* rapid acetylators, *R* rifampin, *RSE* relative standard error*, SA* slow acetylators, *t*_*lag*_ absorption lag time, *V*_*d*_ apparent volume of distribution, *V*_*1*_ apparent volume of the central or plasma compartment in a two-compartment model, *V*_*2*_ apparent volume of the peripheral compartment in a two-compartment model, *Z* pyrazinamide

### PopPK modelling

Majority of the studies employed NONMEM for PopPK modelling (*n* = 9). Two studies used Monolix (Sekaggya-Wiltshire et al. and Horita et al.), and one study employed Phoenix NLME software (Gao et al.) for modelling. Except for the study by Soedarsono et al. who explained the structural model by one-compartment, two-compartment model was used by all the other studies to describe isoniazid disposition characteristics. The disposition of isoniazid was well described using first-order elimination in all the studies, with first-order absorption in most of the studies, except for one study by Cho et al. who explained using sequential zero-order and first-order absorption. The delay in drug absorption was accounted using absorption lag time (*t*_lag_) in studies conducted by Cho et al. Sekaggya-Wiltshire et al. and Naidoo et al. and transit compartments were used by Sundell et al. Denti et al. Panjasawatwong et al. and Abdelwahab et al. The absorption rate (*K*_*a*_) ranged from 0.25 to 3.9 h^−1^ for studies conducted in adult TB patients [25–28, 30–32, 35]. Horita et al. reported a *K*_*a*_ of 4.23 h^−1^ among pediatric TB patients. The apparent volume of the central or plasma compartment in a two-compartment model (*V*_1_) and apparent volume of the peripheral compartment in a two-compartment model (*V*_2_) for all the two compartment models of adult TB patients ranged from 1.5 to 73.4 L and 3.8 to 125.8 L respectively. Whereas, for pediatric TB patients *V*_1_ and *V*_2_ ranged between 3.7 to 16.6 L and 1.07 to 15.3 L, respectively. Higher *V*_1_ and *V*_2_ of 130 and 28.5 L were reported among the pregnant TB patients. The apparent intercompartmental clearance (*Q*) ranged between 1.1 and 43.7 L/h among the PopPK models in adult TB population. The *Q* was 8.46 L/h and 28 L/h for the PopPK models of pediatric TB patients and 12.4 L/h for pregnant TB population. Additive error model was used in three studies (Soedarsono et al. Gao et al. and Panjasawatwong et al.), and proportion error model was used in two studies (Sundell et al. and Huerta-García et al.) to describe the residual variability. A combination of both additive and proportional error models was used in six studies (Cho et al. Sekaggya-Wiltshire et al. Naidoo et al. Denti et al. Horita et al. and Abdelwahab et al.), whereas exponential error model was recorded in one study (Jing et al.) for explanation of residual variability.

### Influence of *NAT2* genotype/SNP on isoniazid clearance

*NAT2* genotype or SNP was tested as a covariate for PopPK model by all the studies. All the studies have reported *NAT2* genotype/SNP as a significant covariate affecting the clearance of isoniazid. Among the seven studies conducted in adult TB population and had *NAT2* genotypes, patients were classified into three categories of rapid, intermediate, and slow acetylators. Two studies (Soedarsono et al. and Jing et al.) reported that the rapid acetylators had more than threefold increase in the isoniazid clearance compared to slow acetylators, with the remaining five studies showing more than twofold isoniazid clearance for rapid acetylators when compared to slow acetylators. Denti et al. reported a comparatively lower fold of increase in isoniazid clearance for combined group of rapid acetylators and intermediate acetylators, when compared to slow acetylators. The range of ratio of isoniazid clearance of *NAT2* rapid acetylators to intermediate acetylators among seven studies conducted in adult TB population who have classified *NAT2* genotype into three categories was 1.3 to 1.9. Among the studies conducted in pediatric population, Panjasawatwong et al. and Horita et al. reported a 2.2- and 1.8-fold isoniazid clearance for combined rapid acetylators and intermediate acetylators, when compared to slow acetylators. Abdelwahab et al. reported more than threefold increase in the isoniazid clearance of *NAT2* rapid acetylators compared to slow acetylators among the pregnant TB patients. The bar chart representing the isoniazid clearance among the three *NAT2* genotypes across the 11 studies is shown in Fig. 2.

### Influence of other covariates on pharmacokinetic variables of isoniazid

Anthropometric measurements such as body weight [25, 26, 28, 29, 31, 34, 35], lean body weight [27], and free-fat mass [32, 33, 36] were reported to affect the isoniazid clearance. Other covariates influencing isoniazid clearance was coadministration of efavirenz in TB patients with HIV [31] and post menstrual age among pediatric TB population [34]. The equation describing the influence of all these covariates on isoniazid clearance is shown in Table 3. Gender and CD4 cell count were reported to affect isoniazid bioavailability among patients co-infected with TB and HIV [29]. Body mass index was a significant covariate affecting apparent volume of the central or plasma compartment in a two-compartment model (*V*_1_) [30]. Different fixed dose combination (FDC) tablet formulations of ATT affected the bioavailability and absorption of isoniazid [31].

### External validation

Only Gao et al., Cho et al., and Huerta-García et al. studies had externally evaluated the developed PopPK model [26, 27, 30].

### Study bias

A potential source of bias involved in this systematic review is the differences in the SNPs panel used for inferring *NAT2* genotype. Additionally, diverse genotyping methodologies for screening *NAT2* genotyping were used in different studies.

## Discussion

### Available PopPK studies on isoniazid

The first PopPK model for isoniazid was described way back in 1997 by Peloquin et al. A one-compartment open model with first-order absorption and elimination was used to describe isoniazid disposition characteristics. The study involved the administration of a single dose of 250 mg of isoniazid to 24 healthy male volunteers and who did not have *NAT2* genotype [37]. Since then, several PopPK studies on isoniazid, with and without *NAT2* genotype as a covariate have been carried out in both TB patients and healthy populations. Most of the studies have used a parametric approach for building the PopPK model for isoniazid. Few studies employing nonparametric approach for isoniazid PopPK have been excluded from the current study to prevent disparities in the comparison [38–40]. Except for Panjasawatwong et al. which employed blood and cerebrospinal fluid (CSF) sampling in the Vietnamese pediatric TB meningitis population, all the other studies employed blood sampling for isoniazid level estimation. Unlike many other ATT, therapeutic drug monitoring (TDM) of isoniazid by saliva sample is not a viable alternative to blood sample [41]. Most of the isoniazid PopPK studies with *NAT2* genotype/SNP have been reported from high TB burden World Health Organization (WHO) regions of South-East Asia and Africa (Asian, *n* = 5; African *n* = 6). The eight countries that account for two-thirds of the global TB cases include India (26%), China (8.5%), Indonesia (8.4%), the Philippines (6.0%), Pakistan (5.8%), Nigeria (4.6%), Bangladesh (3.6%). and South Africa (3.3%) [3]. Our review identifies a significant dearth of *NAT2* genotype-based isoniazid PopPK studies from several high TB burden Asian countries, western populations, and pediatric and pregnant TB populations.

WHO had recommended a daily adult dose of 5 (range: 4–6) mg/kg for isoniazid [42]. Despite intake of weight band-based dose of isoniazid, we identified significant variations in the isoniazid disposition characteristics within a study population and across various studies, suggesting the need for more population-specific isoniazid PopPK models for providing precision therapy of isoniazid. Several isoniazid PopPK models suggest higher isoniazid doses than the conventional weight-based isoniazid doses, particularly among rapid acetylators [39, 43]. *NAT2* genotype-based PopPK models have recommended highly variable doses across different populations. Cho et al. suggested isoniazid once-daily doses of 400, 300, and 200 mg for rapid, intermediate, and slow *NAT2* acetylators, respectively, for Korean TB patients [27]. Whereas, Jing et al. recommended daily isoniazid doses of approximately 800 mg, 500 mg, and 300 mg for rapid, intermediate, and slow *NAT2* acetylators, respectively, among Chinese TB patients [28].

Several studies did not have information regarding the co-administration of drug(s). Physiologically based pharmacokinetic (PBPK) analysis has shown the likelihood of DDIs between CYP2C19 and CYP3A4 substrates with isoniazid, more likely among *NAT2* slow acetylators [44]. TB patients have been reported to have a considerable number of clinically important potential DDIs (pDDIs) [45]. As co-administration of another drug with isoniazid may represent a potential covariate influencing the isoniazid drug levels, we recommend further isoniazid PopPK studies to include this covariate for testing in the model building approach. Limited studies have explored the potential effects of comorbidities such as HIV infection and DM on isoniazid PopPK. Lower exposures of isoniazid were reported in TB patients with these comorbidities [43, 46, 47]. Hence, future PopPK modelling studies may involve testing these comorbidities, along with several others, such as cardiovascular diseases, epilepsy, anemia, vitamin deficiencies, asthma/COPD, and the drugs used in their management as covariates for the development of isoniazid PopPK model. The pharmacokinetic parameters were reported to be comparatively less in non-fasting TB patients when compared to TB patients who were in a fasting state when isoniazid was taken [48, 49]. Few studies have reported whether the patient had taken the drug in a fasting/or a non-fasting state. This information may be potentially valuable when the sample taken is pre-dose of isoniazid. However, when subsequent samples are taken further, information regarding the extent/duration of the fasting state after intake of isoniazid, along with the type of food taken after intake of a dose, may be required to arrive at more conclusive evidence of the effect of fasting state and type of food on isoniazid exposure.

Except for one study [25], all the *NAT2* genotype/SNP-based isoniazid PopPK studies have described isoniazid disposition characteristics by a two-compartment model, as shown in Table 3. An isoniazid PopPK study conducted among the Indian pediatric TB population also described isoniazid disposition by one-compartment model [50]. Significant variabilities in CL/*F*, *V*_1_, *V*_2_, *K*_*a*_ and *Q* estimates were observed across the studies, highlighting the need for population and individual specific *NAT2* genotype-derived isoniazid PopPK model for precise dose calculation. We recommend future studies, particularly with larger sample sizes, divide the sample population into two groups, one for model development and another for model validation. External validation would aid in ensuring the generalizability and reproducibility of the model before its implementation in clinics or policy making.

### *NAT2 *SNPs/genotype and its influence on isoniazid disposition

Isoniazid is primarily metabolized (50–90%) by the NAT2 enzyme encoded by the *NAT2* gene [51]. The seven commonly occurring SNPs in the coding region of the *NAT2* gene represented as codon change at nucleotide position (rs identification; amino acid change in NAT2 protein) are G191A (rs1801279; Arginine64Glutamine), C282T (rs1041983; Tyrosine94Tyrosine) T341C (rs1801280; Isoleucine114Threonine), C481T (rs1799929; Leucine161Leucine), G590A (rs1799930; Arginine197Glutamine), A803G (rs1208; Lysine268Arginine), and G857A (rs1799931; Glycine286Glutamic acid) [52]. SNPs in the *NAT2* gene can affect the structural and functional effects of NAT2 enzymes by altering the size and shape of the active site pocket/conformational changes, reducing catalytic activity and protein stability, and enhancing protein degradation [53–55]. The *NAT2* acetylator genotypes classified as rapid, intermediate, and slow are derived from *NAT2* haplotypes, which are derived from the SNP combinations [56]. Consequently, these *NAT2* acetylator genotypes account for the trimodal isoniazid elimination pattern as shown in Table 3 and Fig. 2. The most common SNP panel that was used for deriving the *NAT2* genotype was the six SNP panels of c.282C > T, c.341 T > C, c.481C > T, c.590G > A, c.803A > G, and c.857G > A. SNPs at c.341 T > C, c.590G > A, and c.857G > A of the *NAT2* gene were investigated by all the studies that derived *NAT2* genotype, except for a study which had investigated only one SNP. We recommend future isoniazid PopPK studies to assess the *NAT2* genotype, including six or seven SNP panels with a specific focus on SNP investigations at c.341 T > C, c.590G > A, and c.857G > A. This provides more insights into the impact of genotype on the pharmacokinetics of isoniazid. Pharmacogenomic-guided therapy (PGT) of isoniazid could represent a cost-effective strategy for managing TB in countries such as Brazil, South Africa, and India [57]. *NAT2* genotype was a significant covariate influencing the pharmacokinetics of isoniazid in all the studies. Most of the studies reported a trimodal clearance pattern for isoniazid to the three *NAT2* genotypes. Two pediatric and one adult TB population studies combined fast and intermediate acetylator phenotypes and reported isoniazid clearance as a bimodal pattern. Studies have shown to have significant isoniazid clearance differences between rapid and intermediate *NAT2* acetylators. Therefore, we recommend the classification of *NAT2* genotype into three categories and consequent description of isoniazid clearance in TB patients in a trimodal pattern for more precise calculation of isoniazid dosage regimen.

### Isoniazid pharmacokinetic-pharmacodynamic studies

Isoniazid clearance directly determines the isoniazid AUC_0-24_ of each patient, and the AUC_0-24_ values influence the specific degree of bactericidal activity [58]. In vitro model studies have shown that AUC_0-24_/minimum inhibitory concentration (MIC) ratio was the pharmacokinetic-pharmacodynamic index that well described the bactericidal activity of isoniazid [58, 59]. In a pharmacokinetic-pharmacodynamic model derived from a longitudinal cohort study conducted among Malawian drug-sensitive TB patients on standard therapy, higher isoniazid exposure correlated with increased bacillary clearance from sputum during the first 2 months of ATT. Higher isoniazid *C*_max_, *C*_max_/MIC, and AUC_0-24_/MIC correlated with treatment success at the end of treatment [60]. Simulation results from a combined PBPK/pharmacodynamic model suggest that rational adjustment of isoniazid doses requires consideration of the regional prevalence of *NAT2* acetylator status for increasing the treatment efficacy and reducing the probability of adverse events, treatment failure, and the emergence of drug resistance [61]. A pharmacokinetic-pharmacodynamic model assessing relationship between drug exposure and survival among adult TB meningitis patients reported that isoniazid exposure was associated with survival. Isoniazid exposures had a bimodal distribution, with the estimated clearance of slow and fast metabolizers were 18.1 L/h and 40.7 L/ respectively. The median AUC of slow and fast metabolizers were 24.2 and 8.9 h*mg/L, respectively. Lower isoniazid exposure was predictive of death and linked to the fast metabolizer phenotype, suggesting that isoniazid doses ≥ 10 mg/kg/day should be investigated in fast acetylator adult TB meningitis population [62]. Donald et al. reported that homozygous slow acetylator and homozygous fast acetylator pulmonary TB (PTB) patients required 3 mg/kg (150 mg for a 50-kg patient) and 6 mg/kg (300 mg for a 50-kg patient) dose, respectively to achieve a satisfactory isoniazid exposure to assure optimal bactericidal activity [63]. Simulations from the pharmacokinetics-pharmacodynamics model of phase 2A AIDS Clinical Trials Group (ACTG) A5312/INHindsight study revealed that 10 and 15 mg/kg doses of isoniazid are required for *inhA*-mutated isolates in slow and intermediate *NAT2* acetylators, respectively, to achieve a drop in bacterial load comparable to 5 mg/kg against drug-sensitive tuberculosis, whereas *NAT2* fast acetylators underperformed even at doses of 15 mg/kg [64]. Higher isoniazid concentration was associated with a faster time to culture conversion among adult PTB patients with DM [46]. Hence, it is evident that clinical outcome and bactericidal activity in TB patients is dependent on isoniazid exposure (AUC_0-24_), which is determined by clearance.

### Influence of other covariates on isoniazid disposition

In all the identified studies, anthropometric variables such as body weight, lean body weight, body mass index (BMI), and fat-free mass were significant covariates affecting the pharmacokinetics of isoniazid. The effect of these variables was accounted for either by standard allometric scaling or by estimation of coefficients. Pregnancy is associated with an increase in fat-free mass [65]. Pregnancy has been reported to increase the clearance of isoniazid by 26% [66]. Abdelwahab et al. had reported a comparatively high isoniazid clearance of 97.1 L/h, 75.7 L/h, and 29 L/h for *NAT2* rapid, intermediate, and slow acetylators, respectively [36]. Further PopPK studies are warranted to assess whether an increase in isoniazid doses is required in the pregnant TB population, particularly among *NAT2* rapid acetylators. We recommend future PopPK studies to assess anthropometric variables and test them as potential covariates to characterize the disposition of isoniazid. In PopPK studies conducted among TB patients with HIV infection, CD4 cell count, gender, efavirenz, and FDC formulation of ATT also influenced isoniazid disposition. Decrease in isoniazid exposure has been previously reported when it was concomitantly administered with efavirenz [67]. Post menstrual age was reported as a significant covariate for the pediatric TB population by Panjasawatwong et al [34]. Previous reports have evidenced the significant role of enzyme maturation for each of the three *NAT2* genotype groups on isoniazid clearance [68].

### Limitations

Most of the studies included in our systematic review did not report the ethnicity of the TB patients. In the absence of definitive information on ethnicity, we stratified the population of each study by country, as shown in Table 2. Significant interethnic and intraethnic variabilities exist in the frequency of SNPs in the *NAT2* gene [69]. Hence, extrapolations of *NAT2* genotype data should not be carried out even within a population. The current study was restricted to articles published in English; therefore, inferences from studies published in other languages are missed. While this review details about the different PopPK models for isoniazid, it does not address the generalizability of these models which might help in using of these models for informed dosing decisions in clinical settings across populations.

## Conclusion

The PopPK modelling approach incorporating several potential covariates including *NAT2* genotype, anthropometric measures, and other clinical variables could provide a thrust for precise optimization of individual dosing regimens of isoniazid in TB patients in this era of precision therapy. All the studies have reported that *NAT2* genotype/SNP was a significant covariate affecting the clearance of isoniazid. Most of the PopPK studies conducted in adult TB patients reported a twofold or threefold increase in isoniazid clearance for *NAT2* rapid acetylators compared to *NAT2* slow acetylators. *NAT2* genotype-based isoniazid PopPK studies are required from several parts of the world, particularly from high TB burden Asian countries and western populations, as well as in pediatric and pregnant TB populations for initiating a pharmacogenomic-guided therapy to TB patients for improving clinical outcomes and reducing adverse drug reactions. Further studies exploring the generalizability of the available models by integrating them or systematic external evaluation could help in identifying the adaptability of models to specific populations across the globe and to facilitate implementation of available models in clinical practice.
